# Anti‐Inflammatory Efficacy of a Functional Shampoo Mixed With 0.063% Artemether for Seborrheic Dermatitis

**DOI:** 10.1111/jocd.70461

**Published:** 2025-09-22

**Authors:** Qin Pei, Yue Yu, Mei‐Lin Huang, Mao‐Lin Zhou, Jian‐Biao Fei, Hai‐Hong Qin

**Affiliations:** ^1^ Department of Dermatology Shanghai University of Medicine and Health Sciences Affiliated Zhoupu Hospital Shanghai China

**Keywords:** anti‐inflammatory therapy, artemether, malassezia, seborrheic dermatitis

## Abstract

**Background:**

Seborrheic dermatitis (SD) is a chronic inflammatory skin disease. Artemisinin and its derivatives have been applied in the treatment of various skin diseases, but they have not yet been used in the treatment of SD.

**Aims:**

To evaluate the therapeutic effect and anti‐inflammatory effect of 0.063% artemether shampoo on SD through a guinea pig model.

**Method:**

Fifty SD‐induced guinea pigs were randomized into control, ketoconazole (2%), matrix, and artemether (0.063%) groups (*n* = 10/group). Treatments were applied twice weekly for 2 weeks. Erythema and scaling were scored pre‐/posttreatment. Lesional IL‐1β, IL‐8, IL‐17, NF‐κB, and histamine H1 levels were quantified via immunohistochemistry.

**Results:**

Artemether group demonstrated lower scores versus control and matrix group (both *p* < 0.05), comparable to ketoconazole group (*p* > 0.05). Artemether group reduced IL‐1β, IL‐8, NF‐κB, and histamine H1 vs. control group(*p* < 0.05), with IL‐8 suppression exceeding ketoconazole and matrix group (*p* < 0.05; *p* < 0.001). No intergroup differences in IL‐17 were observed (*p* > 0.05).

**Conclusion:**

The 0.063% artemether shampoo has comparable clinical efficacy to the 2% ketoconazole shampoo in the treatment of SD, and may exhibit stronger anti‐inflammatory effects.

## 1 Introduction

Seborrhoeic dermatitis (SD) is a chronic, recurrent inflammatory dermatosis predominantly affecting sebaceous gland‐rich regions including the scalp, facial (T‐zone), chest, and intertriginous areas [1]. The condition presents clinically with erythema, greasy scaling, and pruritus [1]. SD manifests across all age groups, with distinct clinical presentations categorized into infantile (self‐limiting) and adult (chronic relapsing) forms [2]. Epidemiological studies reveal age‐related prevalence variations, demonstrating higher incidence rates among middle‐aged and elderly populations [3]. Global prevalence estimates range from 2.35% to 11.3% [4, 5], with particularly high rates observed in North America where scalp involvement affects 17.2% of adults [6]. Beyond its cutaneous manifestations, SD imposes substantial quality‐of‐life impairments and socioeconomic burdens [7, 8, 9]. Contemporary understanding identifies three fundamental prerequisites for SD pathogenesis [2, 10]: colonization by *Malassezia* species, sebaceous lipid overproduction, and host immune hyperresponsiveness. The disease mechanism progresses through five sequential stages [10]: (1) Sebum excretion onto the cutaneous surface. (2) Malassezia colonization of lipid‐rich microenvironments. (3) Fungal lipase‐mediated generation of pro‐inflammatory free fatty acids and lipid peroxides. (4) Immune activation with cytokine cascades driving keratinocyte hyperproliferation. (5) Skin barrier disruption. First‐line topical therapies for facial SD include ciclopirox olamine, ketoconazole, pimecrolimus, tacrolimus, and lithium gluconate/succinate. Common adverse effects associated with these agents include cutaneous irritation, xerosis, and transient erythema [10]. Scalp SD management typically employs 1%–2% ketoconazole shampoo, 2.5% selenium sulphide formulations, or 1%–1.5% ciclopirox solutions [11]. Systemic antifungals are reserved for recalcitrant or generalized cases, though hepatotoxicity and cutaneous adverse events remain concerning [12]. Current therapies demonstrate limited therapeutic scope and poor adherence rates, frequently resulting in disease recurrence [12]. This necessitates the development of multi‐targeted anti‐SD agents with long‐term safety profiles and minimal adverse effects.

Developed following the 1970s isolation of artemisinin from *Artemisia annua L*. to enhance stability [13], artemisinin derivatives (principally artemether and artesunate) demonstrate therapeutic potential in inflammatory dermatoses, including dermatitis [14], rosacea [15], and psoriasis [16], exhibiting favorable safety profiles with minimal adverse effects [17, 18]. SD pathophysiology involves cutaneous colonization by *Malassezia* spp. (mainly *M. restricta, M* *. globosa* ), potential coinfections (*Cutibaterium* and *Staphylococcus*) infections [19], and dysregulated cytokine networks (IL‐1α/β, IL‐2/4/6/8/10/12, TNF‐α, β‐defensin, IFN‐γ) [10]. Artemisinin and its derivatives not only have the effect of anti‐plasmodium, but also possess antiviral, antifungal, antibacterial, and other effects [18]. Notably, artemether inhibits Malassezia growth (MIC: 0.63 mg/mL) [20]. Artemisinin derivatives mediate many signaling pathways modulation—suppressing pro‐inflammatory mediators [21] and upregulating regulatory cytokines (IL‐4/10, TGF‐β) to induce immune tolerance [22]. Experimental studies demonstrate that artemether suppresses the proliferation of HaCaT keratinocytes and concomitantly induces apoptosis in vitro. In addition, it modulates keratinocyte differentiation in a dose‐dependent manner, restoring epidermal homeostasis in vivo [23].

Yet its anti‐inflammatory efficacy and skin barrier repair effect in SD remain unexplored. Artemether may be a multifunctional drug for the treatment of SD. We prepared a shampoo containing 0.063% artemether and applied it to the animal model of SD. This study evaluated the improvement of clinical signs and the anti‐inflammatory effect of artemether in the treatment of SD.

## 2 Materials and Methods

### 2.1 Study Design

Following clearance from the Institutional Ethics Committee Review Board (ethical certificate number: 2025‐03‐10), building on Hirayasu et al.'s SD guinea pig model [24], 40 male Hartley strain guinea pigs at the age of 4 weeks were raised in a sterile environment. 100 μL of olive oil and 100 μL of Malassezia furfur (Strain number: BNCC337308, Beina Chuanglian Biotechnology Institute (Beijing, China)) bacterial suspension were applied continuously for 7 days on the dorsal skin of the guinea pigs. Eventually, erythema and scales appeared in the skin lesion area.

The samples were randomly divided into four group s(each group *n* = 10): the blank control group, the 2% ketoconazole shampoo group, the matrix shampoo group, and the 0.063% artemether shampoo group. The 2% ketoconazole shampoo (Chemical Substance Registration Number: H20000588) was purchased from Nanjing BaiJingYu Pharmaceutical Company Ltd., China. The matrix shampoo was obtained through the emulsification pretreatment of the base stock material (Chemical Substance Registration Number: XF10‐25, Zaoyang Jiele Detergent Company Ltd., China), and the 0.063% artemether shampoo was obtained by adding 63 mg of artemether (Chemical Substance Registration Number: 71963‐77‐4, purity ≥ 98%, Tongtian Biotech Company, Japan) into 100 g of the matrix shampoo and then emulsifying it. The above‐mentioned shampoo were used to clean the skin lesion area for 5 min, then rinsed off and dried. This was done twice a week, with an interval of 3–4 days, for 2 consecutive weeks. The clinical conditions of the skin lesion area before and after treatment were recorded (Table 1).

**TABLE 1:** Classification of seborrheic dermatitis severity.

Grade	Elements of seborrheic dermatitis severity		
	Erythema	Scaling	Lesional extent
0	None	None	None
1	Mild erythema	Thin, flaky scales	1%–20% area
2	Patchy erythema	Thick, localized scales	20%–50% area
3	Confluent erythema	Confluent hyperkeratosis	≥ 50% area

### 2.2 Immunohistochemical Staining of Inflammatory Factors

Paraffin‐embedded skin lesion sections underwent sequential processing: following cooling and three PBS washes, endogenous peroxidase activity was quenched with 3% H_2_O_2_ (30 min, dark). Sections were incubated with primary antibodies (anti‐IL‐1β, anti‐IL‐8, anti‐IL‐17, antihistamine H1, anti‐NF‐κB; 50 μL/section, 30 min RT) followed by PBS rinsing. After overnight PBS equilibration, streptavidin‐biotin complex (SABC) application (50 μL/section, RT) preceded DAB chromogenic development. Counterstaining with hematoxylin, dehydration through graded alcohols, xylene clearance (5 min), and DPX mounting completed specimen preparation. Automated microscopy captured five random fields per section, with Image J software quantifying positive signal intensity via average optical density (AOD) analysis.

### 2.3 Statistical Analysis

The data were analyzed using GraphPad 9.5 statistical software. The results were expressed as mean ± standard deviation (SD). Clinical severity scores underwent two‐way ANOVA evaluation. The AOD value of inflammatory mediators was assessed via one‐way ANOVA. Dunnett's test (treatment vs. control comparisons) and Tukey's test (intertreatment comparisons) were used. Nonparametric alternatives were employed for non‐normally distributed data or heteroscedastic datasets. Pairwise analyses incorporated Dunnett's correction following Kruskal–Wallis testing. Values of *p* < 0.05 were considered statistically significant.

## 3 Results

### 3.1 Analysis of the Efficacy of 0.063% Artemether Shampoo Based on SD Model

**FIGURE 1:**
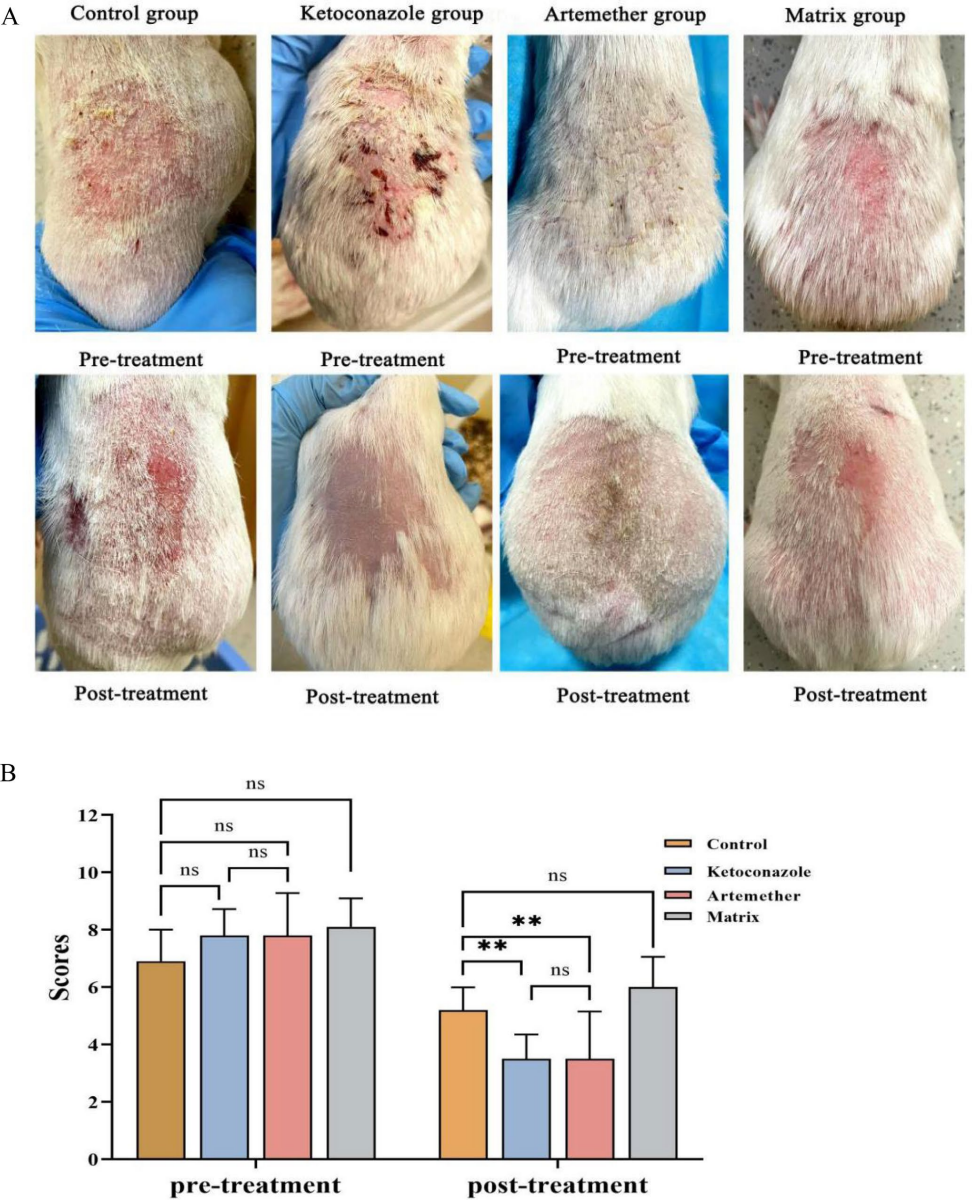
Clinical evaluation of SD in guinea pig model. (A) Comparison of lesional erythema and scaling pre‐ and posttreatment. (B) Comparison of composite clinical scores pre‐ and posttreatment. temporal effects (*p* < 0.001); intergroup variation (*p* < 0.001); time × group interaction effects (*p* < 0.001). Error bars indicate mean ± SD. **p < 0.01, ns, not significant.

Posttreatment analysis revealed significant improvement in lesional parameters (erythema, scaling, lesional extent) across all groups (*p* < 0.001), demonstrating both temporal and therapeutic intervention effects on SD manifestations (Figure 1B). Pretreatment characterization showed extensive scaling and vivid erythema in both ketoconazole and artemether groups, which resolved to minimal residual scaling with complete erythema clearance post‐intervention, accompanied by substantial lesional area reduction (Figure 1A). Both ketoconazole‐ and artemether‐treated groups demonstrated statistically significant reductions in posttreatment clinical scores versus controls (*p* < 0.01). Comparable efficacy between artemether and ketoconazole interventions (*p* > 0.05). There was no therapeutic advantage of matrix formulation over controls (*p* > 0.05). These findings establish parity in therapeutic outcomes between 0.063% artemether and 2% ketoconazole formulations for SD management.

### 3.2 Artemether Significantly Inhibited Inflammatory Factor Expression in SD

**FIGURE 2:**
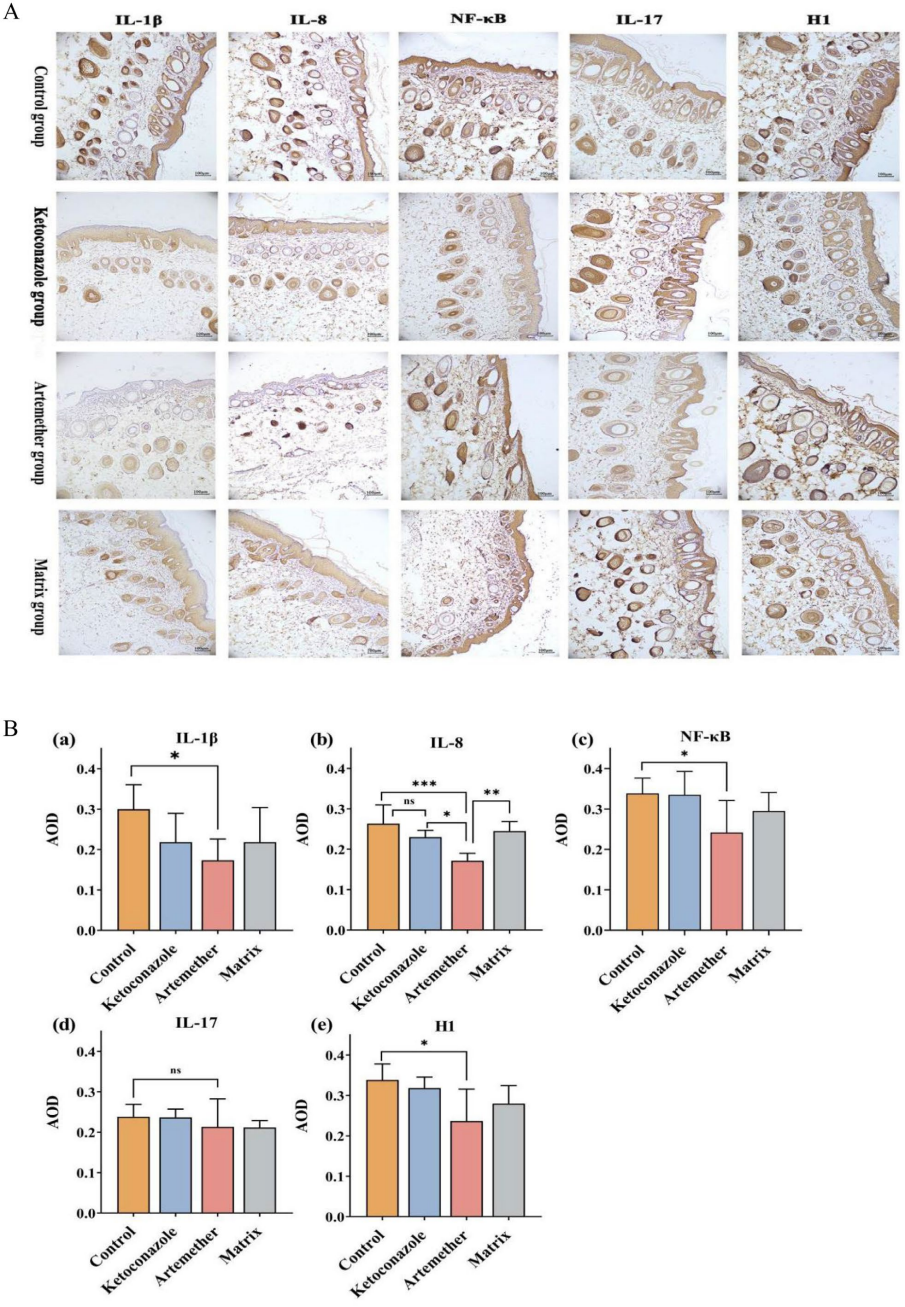
Inflammatory mediator profiling in lesional skin. (A) Representative immunohistochemical staining patterns illustrating expression intensity and spatial distribution of IL‐1β, IL‐8, IL‐17, NF‐κB and H1 receptor across experimental cohorts (scale bar = 100 μm). (B) Quantitative comparison of AOD values for: (a) IL‐1β, (b) IL‐8, (c) NF‐κB, (d) IL‐17, (e) H1 receptor. Error bars indicate mean ± SD. *p < 0.05, **p < 0.01, ***p < 0.001; ns, not significant.

Artemether treatment significantly downregulated lesional expression of IL‐1β, NF‐κB, and histamine H1 compared to controls (*p* < 0.05), paralleled by reduced immunohistochemical staining intensity (Figure 2A). In contrast, ketoconazole and matrix groups showed comparable expression levels to untreated controls (*p* > 0.05). Artemether induced the most pronounced suppression of IL‐8 (*p* < 0.001), while IL‐17 expression remained stable across all groups (*p* > 0.05) (Figure 2B). These findings establish artemether's anti‐inflammatory mechanism in SD through selective targeting of the IL‐1β/IL‐8/NF‐κB signaling axis combined with histamine H1 receptor regulation.

## 4 Discussion

This study is the first to confirm that artemether has an improving effect on SD symptoms. A 14‐day animal experiment showed that 0.063% artemether shampoo has comparable efficacy to 2% ketoconazole and stronger anti‐inflammatory properties, providing foundational data for clinical translation.

The primary factor in the pathogenesis of SD is the heightened proliferation of malassezia, while both artemether and ketoconazole demonstrated antifungal activity through Malassezia suppression [11, 20]. Our results are consistent with the study by Bai et al. [14], suggesting that artemisinin derivatives have the effect of improving dermatitis [14]. In a mouse model of atopic dermatitis, it was observed that intraperitoneal injection of artesunate improved erythematous and scaly lesions and exerted anti‐inflammatory effects [14]. In our study, a safer external washing method was adopted; artemether displayed enhanced anti‐inflammatory potency at one‐thirtieth the concentration of ketoconazole.

The resolution of erythema and scales in the artemether‐treated group might be associated with its inhibition of molecules related to the innate immune response in SD lesions, such as IL‐1β, IL‐8, and the NF‐κB pathway. When Malassezia interacts with keratinocytes, various pattern recognition receptors are activated in susceptible individuals [10]. NOD‐like receptor activation triggers inflammasome assembly and caspase‐1‐mediated IL‐1β maturation [10, 25], a process amplified in SD through Th1‐polarized immunity [10]. When Toll‐like receptor 2 is activated, it induces keratinocyte‐derived IL‐8 secretion, driving neutrophil chemotaxis and NF‐κB activation [10, 26]. Consistent with our findings, previous studies have demonstrated that artemisinin and its derivatives can inhibit the secretion of IL‐1β by macrophages and the related functions of Th1 cells [22] and are involved in the inhibition of the NF‐κB pathway [27].

In SD lesions, severely inflamed ones exhibited scratch marks, sparse or absent hair (Figure 1A). Due to itching and inflammation, telogen effluvium may occur, and in severe cases, even scarring alopecia, such as central centrifugal cicatricial alopecia [28]. Histamine, as an inflammatory factor mediating pruritus [29], was found by Vijayaraghavan et al. to be significantly increased in SD lesions compared with healthy scalp [30], which suggests heightened mast cell degranulation [28]. The artemether‐treated group showed no scratches and no hair loss areas (Figure 1A), which may be related to the ability of artemether to inhibit the expression of histamine H1 receptors. Artemisinin and its derivatives inhibit mast cell function and B lymphocyte secretion of IgE [22, 31] and have described anti‐fibrotic effects in vivo and in vitro models of tissue fibrosis [32].

IL‐17 may play an important role in SD. The murine Mpzl3^−/− SD models demonstrate infiltration of IL‐17‐producing γδT cells [33] and human SD transcriptomes show IL23/Th17 pathway activation [27]. However, our findings indicate that artemether is not significantly associated with T lymphocyte‐mediated immune response molecules (such as IL‐17). However, other studies have demonstrated that artemether possesses immunosuppressive effects on T lymphocytes [34], and artemisinin and its derivatives decrease IL‐17 secretion [21, 22, 31]. This may be attributed to the following reasons: compensatory cytokine regulation in artemether's mechanism, secondary role of IL‐17 in established SD lesions, and species‐specific pathway relevance.

## 5 Conclusion

This trial on the SD animal model can only provide preliminary evidence that the 0.063% artemether shampoo exhibits comparable clinical efficacy to the 2% ketoconazole shampoo, with the former demonstrating stronger anti‐inflammatory effects. Study limitations include sample size constraints and short‐term evaluation. Whether artemether can emerge as a promising dual‐action therapeutic candidate for SD requires comprehensive evaluation of its therapeutic potential and safety in the treatment of SD through large‐scale clinical trials.

## Author Contributions

Q.P.: Primary investigator, designing the study, manuscript writing. M.‐L.Z. and M.‐L.H.: Cultivation and activation of malassezia and established animal modeling. J.‐B.F.: Designing and writing methodology. Y.Y.: Collated and analyzed the data. H.‐H.Q.: Manuscript review, technical recommendations and proofreading.

## Ethics Statement

The current study received Ethics approval from the Animal Ethics Committee of Zhoupu Hospital, Shanghai (2025‐03‐10).

## Conflicts of Interest

The authors declare no conflicts of interest.

## Data Availability

The data that support the findings of this study are available on request from the corresponding author. The data are not publicly available due to privacy or ethical restrictions.
